# P-780. Machine Learning Models for Early Urinary Tract Infection (UTI) Prediction from Electronic Health Records

**DOI:** 10.1093/ofid/ofaf695.991

**Published:** 2026-01-11

**Authors:** Nicholas P Marshall, Fatemeh Amrollahi, Fateme Nateghi Haredasht, Stephen Ma, Manoj Maddali, Amy Chang, Stan Deresinski, Niaz Banaei, Mary Kane Goldstein, Steven Asch, Jonathan H Chen

**Affiliations:** Stanford University, Palo Alto, CA; Stanford University, Palo Alto, CA; Stanford University, Palo Alto, CA; Stanford, Palo Alto, California; Stanford University, Palo Alto, CA; Stanford University, Palo Alto, CA; Stanford Health Care, Stanford, CA; Stanford University School of Medicine, Palo Alto, CA; Stanford University, Palo Alto, CA; Stanford University, Palo Alto, CA; Stanford University, Palo Alto, CA

## Abstract

**Background:**

Antibiotic resistance is a growing global threat, and urinary tract infections (UTIs) are a leading driver of inappropriate antibiotic use. Diagnosing true UTIs at the time of culture order is challenging due to variable symptoms and delayed test results, often leading to over-treatment, resistance, drug-related complications, and increased costs. Missed diagnoses risk progression to severe infection. Predictive models using routinely collected electronic health record (EHR) data offer a promising, real-time solution to support stewardship and early decision-making.

**Table 1:**
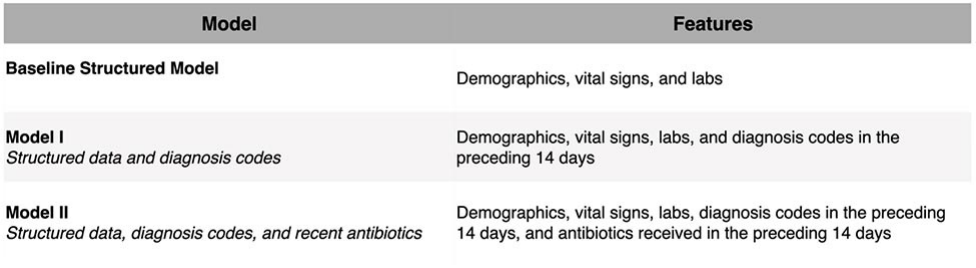
EHR-integrated models feature set description.

**Figure 1:**
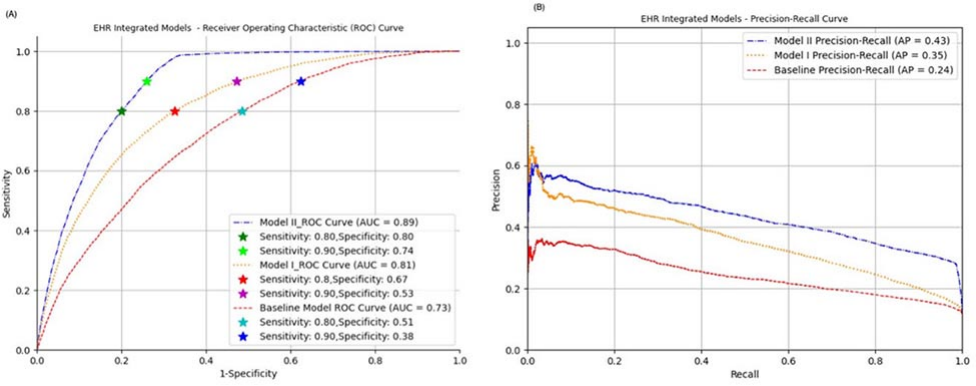
EHR-integrated models’ performance in terms of AUC-ROC and precision-recall curve.

**Methods:**

We developed machine learning models to predict clinical UTIs at the time of urine culture order using structured EHR data. Our dataset included 300,381 urine cultures from 164,327 adult patients at two academic and community hospitals (2015- 2024). A previously validated electronic phenotype (Ma et al., 2024) served as the proxy label, combining microbiologic and treatment criteria. Features included demographics, vital signs, and labs within 24 hours prior to and 2 hours after culture order. Two enhanced models incorporated recent diagnoses and antibiotic use (14 days prior). Models were trained with XGBoost. Performance was assessed on a held-out test set (2023- 2024) using ROC-AUC, NPV, and PPV at sensitivity ≥ 80%.

**Table 2:**
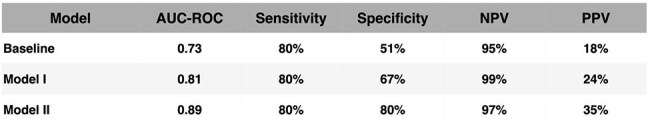
EHR-integrated models’ AUC-ROC, specificity, negative predictive value (NPV), and positive predictive value (PPV) at threshold where sensitivity is greater than or equal to 80%.

**Results:**

The baseline model, using only vital signs and labs, achieved an ROC-AUC of 0.73. At 80% sensitivity, it demonstrated high NPV (95%) but limited specificity (51%) and PPV (18%), making it more useful for ruling out UTIs than confirming them. Adding recent ICD-coded diagnoses (Model I) improved ROC-AUC to 0.81, with better specificity (67%) and PPV (24%), and excellent NPV (99%). Model II, which also included prior antibiotic prescriptions, achieved the highest performance: ROC-AUC 0.89, specificity 80%, NPV 97%, and PPV 35%.

**Conclusion:**

Structured EHR data available at the time of urine culture order can be leveraged to accurately predict clinical UTIs. Incorporating recent diagnoses and antibiotic use significantly enhances performance, enabling scalable, real-time decision support to improve diagnostic precision, guide empiric therapy, and advance antimicrobial stewardship.

**Disclosures:**

Jonathan H. Chen, MD, PhD, Reaction Explorer: Ownership Interest

